# Immunological mechanisms underlying protection mediated by RTS,S: a review of the available data

**DOI:** 10.1186/1475-2875-8-312

**Published:** 2009-12-30

**Authors:** Vasee S Moorthy, W Ripley Ballou

**Affiliations:** 1 Initiative for Vaccine Research, World Health Organization, 1211 Geneva 27, Switzerland; 2 Nuffield Department of Clinical Medicine, John Radcliffe Hospital, University of Oxford, OX3 9DU, UK; 3 Infectious Diseases Development, Global Health Division, Bill and Melinda Gates Foundation, PO Box 23350, Seattle, Washington, USA

## Abstract

The RTS,S/AS candidate malaria vaccine has demonstrated efficacy against a variety of endpoints in Phase IIa and Phase IIb trials over more than a decade. A multi-country phase III trial of RTS,S/AS01 is now underway with submission as early as 2012, if vaccine safety and efficacy are confirmed. The immunologic basis for how the vaccine protects against both infection and disease remains uncertain. It is, therefore, timely to review the information currently available about the vaccine with regard to how it impacts the human-*Plasmodium falciparum *host-pathogen relationship. In this article, what is known about mechanisms involved in partial protection against malaria induced by RTS,S is reviewed.

## Background

Against a background of variably shifting malaria disease burden and a scale-up in the implementation of artemisinin-based combination therapy, long-lasting insecticidal nets and in some settings, indoor residual spraying, *Plasmodium falciparum *malaria remains the commonest cause of under-five mortality in several countries[1]. After four decades of malaria vaccine development, a pivotal phase III trial is underway of a vaccine which may be suitable for licensure and assessment for implementation in malaria-endemic countries. This vaccine, RTS,S/AS, is based on the hepatitis B surface antigen virus-like particle (VLP) platform, genetically-engineered to include the carboxy terminus (amino acids 207-395) of the *P. falciparum *circumsporozoite (CS) antigen[2]. The hybrid malaria-hepatitis B VLP is lyophilized and undergoes point-of-use reconstitution with GlaxoSmithKline's AS01 adjuvant, a mixture of liposomes, MPL and QS21[3]. RTS,S has demonstrated clinical efficacy against both infection and clinical malaria in several well-designed phase II field efficacy trials in both adults and children, replicated at several trial sites [4-7]. The considerations of generalizability of efficacy in different geographic and transmission settings, duration of efficacy and confirmation of efficacy against severe malaria are all to be addressed in the phase III trial[8]. A large database will also be available to provide information on safety of the novel adjuvant AS01E.

Here, the available evidence is re-assessed from clinical trials of the relationships between parasite biology, vaccine-induced immune responses and efficacy for circumsporozoite (CS) -based malaria vaccines.

## Localization and functions of CS protein

What is known about the role of the CS protein in malaria parasite biology and pathogenesis has been reviewed previously[9,10]. Initially identified as a *Plasmodium berghei *ortholog antigen Pb44, the CS protein[11] was shown to be the target of protective antibodies to the sporozoite surface in murine models over 25 years ago [12-14]. CS covers the entire surface of sporozoites[15], the form of the malaria parasite inoculated into humans by female anopheline mosquitoes, and is found on the plasma membrane of liver-stage parasites, which develop after sporozoite invasion of hepatocytes. CS has been detected in the cytoplasm of infected hepatocytes and a recent report indicated that CS plays a role in suppression of liver-stage inflammatory responses in a *P. berghei *model[16]. CS is secreted at the apex of sporozoites, becomes an integral component of the plasma membrane and is continuously released in large amounts at the distal tip of the sporozoite during gliding motility[17,18].

Many observations point to a region of CS as one of the key ligands for adherence to the heparan suphate proteoglycan components of the liver sinusoidal lining prior to hepatocyte invasion[10]. Incubation of live sporozoites *in vitro *with anti-CS antibodies induces a characteristic morphological change in sporozoite appearance with cessation of motility and shedding of sporozoite surface material. This change, dubbed the circumsporozoite precipitin reaction, was first reported with antibodies raised by irradiated sporozoite immunization[19,20], and later with antibodies raised through immunization with only the conserved Asparagine-Alanine-Asparagine-Proline (NANP) amino acid repeat sequence which forms the immunodominant B-cell epitope from *P. falciparum *CS antigen[15]. This sequence is species-specific, but highly conserved for isolates from each species.

## Clinical trial immunogenicity and efficacy

CS-based malaria vaccine development has progressed through iterations using clinical challenge model efficacy as a means of guiding improvements to vaccine design [21-27]. The story of this iterative development in the late 1980s and 1990s, leading up to selection of RTS,S for field trials, is well documented including several review publications. Interested readers are referred to these reviews[2,28-30]. RTS,S/AS01 induces very high IgG concentrations in vaccinated humans to the NANP CS repeat. In addition, this vaccine induces moderate to high CD4+ Th1 responses against flanking region peptides[31].

Immune correlates of protection are known to exist for some vaccines and these permit licensure of new forms of these vaccines and extension of vaccine indications to new populations based on immunogenicity endpoints without a requirement to demonstrate vaccine efficacy (reviewed in [32]). In the case of malaria vaccines, there is no known link between immunogenicity and protection and, therefore, no accepted in vitro correlates of protection[33]. Moreover, the parasite is complex with multiple antigens that are potential targets of naturally acquired immunity. The CS antigen is not thought to be an important target of naturally acquired immunity by individuals repeatedly exposed to malaria-infected mosquitoes.

Analysis of association between immune responses and clinical efficacy have limited utility where the sample size is small, unless the relationship is simple and generalizable amongst vaccinees. Nevertheless, the available experimental human sporozoite challenge trial data for RTS,S with both AS01 and AS02 adjuvants is consistent with an important role of anti-NANP IgG in protection from infection. Each of the challenge studies were necessarily small, with generally too few protected individuals to usefully explore this relationship, in more than an indicative manner[25]. It should be noted that even life-long exposure to large numbers of malaria-infected mosquitoes rarely induces an anti-CS antibody response in excess of 10 μg/mL[34,35] using a qualified ELISA where the capture antigen consists of NANP repeats, and in infants and children living under these conditions, the values rarely exceed 0.5 μg/mL[36].

It has not proved possible to derive a protective threshold for CS repeat IgG concentration, although, should a threshold exist, it probably lies above 20 μg/mL for most individuals: in 10 challenge trials of CS vaccines conducted between 1986 and 2001, only five of 108 volunteers with CS repeat IgG levels below 20 μg/ml were protected, whereas 14 of 27 volunteers with IgG levels above 20 μg/ml were protected[37]. A phase IIa trial of RTS,S/AS02 in 41 vaccinees reported a statistically significant increase in the seroconversion rate above this 20 μg/ml value for protected vaccinees compared to unprotected volunteers[37]. However, in the same trial there was no trend for protection with increasing IgG levels and receiver operator characteristic analyses did not identify a cut-off level for protection. In an analysis of 19 RTS,S/AS02 vaccinees, in a later phase IIa trial, those protected had higher CS repeat IgG levels than those unprotected[38]. None of the vaccinees with an IgG level below 20 μg/ml were protected; some vaccinees with levels above 20 μg/ml were not protected. In a further phase IIa trial of RTS,S/AS02 with 40 vaccinated volunteers, an analysis was performed dividing the vaccinees into three groups: those completely protected from infection, those with a delay in time to first detection of parasitaemia by microscopy, indicating partial protection, and those not protected[39]. In this analysis, the protected group (n = 16) had a geometric mean antibody concentration of 113.7 μg/ml. The equivalent figures for the partially protected (n = 14) and unprotected (n = 8) groups were 67.5 μg/ml and 29.6 μg/ml. These differences were statistically significant[19], and the absolute peak IgG concentrations induced in immunized protected volunteers are clearly very high. The largest Phase IIa trial of RTS,S confirmed the strong association between anti-CS IgG titre and protection against infection and demonstrated an independent, albeit weaker, association between CS- specific CD4+ T cell responses and protection[31]. This trial has not reported on potential threshold levels to date.

The first Phase IIb field efficacy trial, which involved 306 Gambian adults, reported 34% efficacy against the incidence rate of first blood stage infections over a 15-week period. In this study a linear relationship was found between IgG concentration post dose 3 and protection from blood stage infection, such that the odds ratio for a ten-fold increase in IgG concentration was 0.21 (p = 0.023). After correction for age and pre-vaccination titre the odds ratio was 0.27 (p = 0.07). There is some evidence of naturally acquired immunity to infection as detected by microscopy occurring in adolescence and adulthood. For example, in two adult vaccine efficacy trials with primary infection endpoints, the incidence of infection decreased with increasing age in the 18-45 year age range[4,40] and a decrease in parasite prevalence over this age range is well documented in several studies from Kilifi, Kenya[41]. Given the very substantially greater data on naturally acquired immunity targeting the blood stages of the parasite compared to the pre-erythrocytic stages, it may be that this naturally acquired immunity to infection is in fact a gradual acquisition of the ability of anti-blood stage immunity to suppress blood stage infection to sub-microscopic parasite densities rather than sterilising pre-erythrocytic immunity. This muddies the water to some extent with regard to the question of whether vaccine or naturally acquired responses account for protection from infection in studies in older children and adults. Nevertheless, the best chance of detecting relationships between immune responses and protection for pre-erythrocytic vaccines is in field trials in endemic populations with similarly low pre-existing antibodies reflecting prior exposure to malaria, primary infection endpoints and, where the entomological inoculation rate is high, pre-treatment of volunteers prior to the efficacy follow-up period. Here the efficacy endpoint is as close as possible to a likely biological target of the immune response.

Questions remained as to whether this relationship between anti-CS IgG and protection against infection would hold in younger children or infants. It also remained to be seen whether a similar relationship might have been seen between IgG concentration and morbidity endpoints. This would introduce a further variable which is difficult to assess because there may not be a direct relationship between infection and disease: not all infections become clinically manifest, it may not be possible to link a specific clinical case to a specific infectious event, and some mild cases of clinical disease may not be detectable. It is likely that all cases of severe morbidity episodes are detected in field trials, but the still poorly understood heterogeneity in risk of malaria introduces major complexities in extrapolating from infection to morbidity at the individual level. Thus, a lack of association between immune responses and anti-morbidity efficacy would not necessarily be surprising. Furthermore exposure may not be uniform and this has been shown to make immunity harder to detect[42].

The largest Phase IIb field efficacy trial of RTS,S/AS02 to date reported data on 2,022 Mozambican children aged 1-4. In a commendable attempt to address the issue of efficacy against both infection and clinical disease, two separate cohorts were utilised. In one cohort (cohort 1), passive case detection only was performed, without pre-treatment, in order to assess efficacy against clinical disease. In cohort 2, children were pre-treated and active detection of infection was performed with regular cross-sectional blood sampling. Clinical malaria efficacy over 18 months after dose 3 in cohort 1 was 35.3%. There was an unexpectedly high rate of severe malaria disease detected during the study, allowing an estimation of vaccine efficacy against severe malaria of 48.6%[5,6]. A recent paper reports for the first time on the association between anti-NANP IgG and infection efficacy in cohort 2 of this same trial[43,44]. Again there is a statistically significant association between IgG concentration and efficacy against infection. A similar association was reported in an infant RTS,S Mozambican study [45]. In contrast two paediatric randomized controlled field trials have now reported a lack of association between the anti-NANP IgG concentration and protection against clinical disease; cohort 1 of the Mozambican study in children aged 1-4 and a trial conducted in Kenya and Tanzania in children aged 5-17 months[5,7].

What can be deduced from the consistent pattern of associations seen for anti-NANP IgG and protection from infection with RTS,S, and the lack of association with morbidity to date? Efficacy against morbidity should be a natural extension of efficacy against infection in that by reducing the incidence of new infections, reducing the multiplicity of infection and a reduction in parasite density of breakthrough infections should logically be expected to interact with the expression of clinical malaria disease and the acquisition of naturally acquired immunity. One hypothesis proposed here is that this naturally acquired blood-stage immunity component which cannot be directly measured confounds analyses seeking to measure an association of immune responses against the sporozoite with morbidity endpoints which involve a completely different stage of the parasite that does not share protective epitopes with the CS protein.

It should be noted that little is known about the fine specificity or functional activity of anti-NANP IgG and their role in protection. Where it has been analysed, IgG1 and IgG2 account for "nearly all" of the total IgG concentration of anti-NANP antibodies[25], with IgG1 being "the dominant subclass"[37]. Sporozoite-opsonizing activity has been demonstrated *in vitro*, where monocytes have been shown to internalize and kill live sporozoites exposed to plasma from RTS,S-immunized protected vaccinees[46]. Further characterization of the associations between functional activities of anti-NANP IgG and protection is highly desirable and may inform refinements planned for future CS-based vaccine candidates.

Vaccines may protect either through complete protection of a proportion of vaccinees or through partial protection of all vaccinees (or a combination of the two)[47]. Certain characteristics of clinical efficacy data may point to one or the other mode of vaccine effect. The RTS,S challenge and field trial data to date are consistent with at least partial protection in most or all volunteers. It is possible that complete protection also occurs in some volunteers. It has been established that RTS,S/AS reduces the rate of new blood stage infections[5], reduces the initial inoculum of each blood stage infection[48] and reduces the multiplicity of infection[49] in vaccinees. Taken together these may also foster acquisition of naturally acquired immunity to malaria, whilst reducing the malaria morbidity in vaccinees. This mode of action has been called a "leaky vaccine". If RTS,S is indeed a leaky pre-erythrocytic malaria vaccine, this needs to be taken into account in interpreting associations of immune responses and efficacy, as partial protection from infection would be expected in most individuals. Recent advances in understanding of the skin stage of malaria, help us envisage how such partial protection could occur. When a mosquito probes for a blood meal, sporozoites are deposited intradermally and migrate for up to two hours before entering skin microvasculature or entering lymphatics[50]. However, there is a wide range of skin transit times before sporozoites enter the vasculature[51], with some sporozoites perhaps entering directly into vessels during mosquito probing. Anti-sporozoite antibodies have been shown to reduce the numbers of sporozoites which enter skin blood vessels to begin the journey to the liver[52].

What is the role of cell-mediated immune (CMI) responses in protection afforded by RTS,S? The published literature indicates that there is evidence that CMI has an important role when added to the foundation of robust IgG responses. CMI indicators were used as a down-selection criterion for adjuvant choice in the RTS,S programme[2]. Both CS-specific γ-interferon secreting CD4+ T cell responses (as enumerated by *ex vivo *ELSISPOT) and multifunctional CS-specific CD4+ T cells (defined as expressing two or more of γ-interferon, TNF, IL-2 and CD40 ligand using an intracellular cytokine staining assay) were greater in protected than in unprotected vaccinees in a recent RTS,S clinical challenge trial[31]. Multifunctional CD4+ T cell responses were reported not to be correlated with anti-NANP IgG responses. There are major limitations in what CMI studies are possible with blood volumes obtainable in paediatric trials, although some data on CMI responses to RTS,S is now available in African children[53]. Most RTS,S studies performing CMI studies have reported an absence of substantial CS-specific CD8+ T cell responses[31,54]. Weak CS-specific CD8+ T cell responses were reported in 1 study with a highly sensitive ELISPOT assay performed on cultured cells[55].

## CS-specific CMI and vaccine efficacy

RTS,S-induced CS-specific CD4+ T cell frequency, as enumerated by both *ex vivo *ELISPOT and intracellular cytokine staining, is associated with protection against infection[31]. However the available data indicates that IgG plays a more important role in RTS,S-mediated protection than CMI. The potential contribution of CD8+ T cells in killing of intracellular hepatocyte infection is unquestioned. This evidence stems from adoptive transfers and pre-clinical models of whole organism and subunit vaccine immunity [56-58] with some indirect evidence from clinical studies[59,60]. CD8 T cells are thought to be the critical determinant of irradiated sporozoite immunity, at least in mouse models where immune mechanisms can be dissected in detail[58]. There is, therefore, good reason to believe that induction of robust liver-stage specific CD8+ T cell responses in addition to RTS,S-induced IgG and CD4+ T cells, could add to currently achieved levels of clinical protection. There is also evidence for the important role of CD4+ Th1 responses from both pre-clinical adoptive transfer experiments[61] and field trials[62]. Furthermore protective CD8+ responses may be CD4+ T cell dependent[58]. Thus further improving upon currently attained CD4+ T cell magnitude may augment RTS,S-induced protection[63]. It is worth noting that modest CD4+ T cell *ex vivo *γ-interferon responses alone at an arithmetic mean of 129 spot forming cells/million PBMCs induced by DNA/modified vaccinia virus Ankara prime-boost delivery of *P. falciparum *CS were not associated with efficacy in a sporozoite challenge trial in the UK[64].

## Conclusions

**Figure 1:**
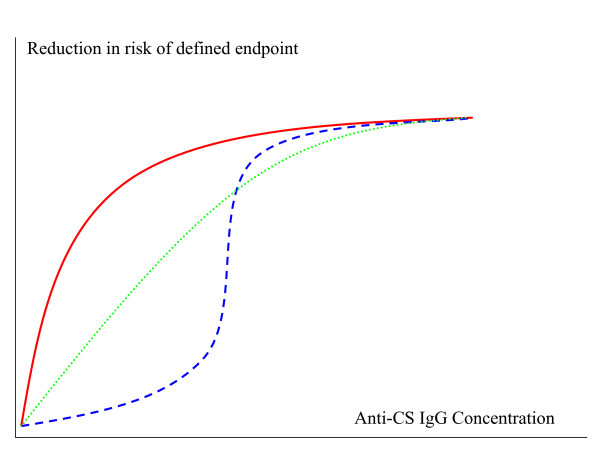
**The relationship between a protective immune response and reduction in risk of a defined malaria endpoint**. If the risk of a defined malaria endpoint (infection or disease) is inversely related to a vaccine-induced immune response, it may be possible to describe this relationship graphically. Three hypothetical relationships are shown above. The blue dashed line represents a relationship for which a clear threshold could be calculated using efficacy trial data, but in a biological system such a relationship is perhaps less likely than the dotted green and solid red lines.

The available evidence about the protective mechanism of RTS,S/AS strongly supports a critical role for IgG against the CS repeat sequence in the protection seen against infection, whether in multiple clinical challenge trials in USA, adult or paediatric field trials in different age groups and across the distinct transmission settings of The Gambia, Kenya, Tanzania and Mozambique. Two conclusions follow from this fact. Firstly, future attempts at improvements of RTS,S-mediated protection should be rooted in at least matching the potent IgG response. Secondly, exploratory studies to shed some light on the fine specificity and protective mechanism of the IgG response are a high priority. These same data do not support identification to date of an absolute correlate of protection in the sense of a threshold level where complete protection is conferred at the individual level against a defined endpoint, but this lack of an absolute correlate does not change the above conclusions. The relationship between a protective immune response and reduction in risk of a defined malaria endpoint, be it infection or clinical disease, could perhaps be described graphically (see Figure 1). This type of representation of correlates of immunity has been performed for some other diseases[32] and it may be beneficial for this sort of analysis to be attempted for RTS,S-induced immune responses and malaria efficacy.

There is supportive evidence, although weaker than that for the role of antibodies, that CS-specific CD4+ T cell responses are independently associated with protection against infection. A parsimonious interpretation is that such CD4+ T cell responses and IgG are additive in their protective effect for RTS,S. In some individuals a protective effect of moderate antibody concentrations may be complemented by a strong T cell response and vice versa. From this it follows that second generation vaccines that are able to match the protective B cell response seen in RTS,S vaccinees, but improve on the CMI aspect, would have a good chance of inducing higher efficacy. A valid hypothesis for vaccine approaches, which induce CMI responses without antibody induction, is whether spectacular CMI responses an order of magnitude higher than has been seen to date with CS-based vaccines could protect in the absence of antibodies. Though an interesting research question, this is a higher risk approach than dual induction of potent IgG and CMI.

Given the recent demonstration of partial efficacy in humans through prime-boost immunization with the ME-TRAP construct in the UK, two approaches appear highly worthy of attention from a technical perspective. These are matching the potent IgG response induced by RTS,S, and improving upon either the malaria-specific CD8+ or the CD4+ T cell responses.

## Competing interests

WRB is an inventor on patents in the field of malaria vaccines. VSM declares that he has no competing interests.

## Authors' contributions

VSM conceived the idea for the article and wrote the first draft of the manuscript. WRB contributed significantly to the writing of the manuscript. Both authors read and approved the final manuscript.
